# Trichostatin A Promotes the Generation and Suppressive Functions of Regulatory T Cells

**DOI:** 10.1155/2013/679804

**Published:** 2013-05-08

**Authors:** Cristian Doñas, Macarena Fritz, Valeria Manríquez, Gabriela Tejón, María Rosa Bono, Alejandra Loyola, Mario Rosemblatt

**Affiliations:** ^1^Departamento de Ciencias Biológicas, Universidad Andrés Bello, República 275, Santiago, Chile; ^2^Fundación Ciencia & Vida, Avenida Zañartu 1482, Ñuñoa, Santiago, Chile; ^3^Laboratorio de Inmunología, Departamento de Biología, Facultad de Ciencias, Las Palmeras 3425, Ñuñoa, Universidad de Chile, Santiago, Chile; ^4^Universidad San Sebastián, Avenida Lota 2465, Providencia, Santiago, Chile

## Abstract

Regulatory T cells are a specific subset of lymphocytes that suppress immune responses and play a crucial role in the maintenance of self-tolerance. They can be generated in the thymus as well as in the periphery through differentiation of naïve CD4^+^ T cells. The forkhead box P3 transcription factor (Foxp3) is a crucial molecule regulating the generation and function of Tregs. Here we show that the *foxp3* gene promoter becomes hyperacetylated in *in vitro* differentiated Tregs compared to naïve CD4^+^ T cells. We also show that the histone deacetylase inhibitor TSA stimulated the *in vitro* differentiation of naïve CD4^+^ T cells into Tregs and that this induction was accompanied by a global increase in histone H3 acetylation. Importantly, we also demonstrated that Tregs generated in the presence of TSA have phenotypical and functional differences from the Tregs generated in the absence of TSA. Thus, TSA-generated Tregs showed increased suppressive activities, which could potentially be explained by a mechanism involving the ectonucleotidases CD39 and CD73. Our data show that TSA could potentially be used to enhance the differentiation and suppressive function of CD4^+^Foxp3^+^ Treg cells.

## 1. Introduction

Regulatory T cells (Treg) are a specific subset of lymphocytes that suppress immune responses and play a crucial role in the maintenance of self-tolerance [1, 2]. Their development and function are programmed by the forkhead box P3 transcription factor Foxp3, which is predominantly expressed in CD4^+^CD25^+^ Treg cells [1, 3]. Tregs actively suppress the activation and expansion of autoreactive immune cells to limit the duration and extent of inflammation. Therefore, a decrease in Treg activity can contribute to autoimmunity and inflammatory diseases [4]. Because of their suppressive capacities, Tregs represent a promising strategy for inducing tolerance to self- and non-self-antigens in such diseases.

In recent years, increasing evidence has demonstrated the role of epigenetic alterations in the etiology of many autoimmune and inflammatory diseases through changes in DNA methylation and histone modifications [5, 6]. Therefore, it is important to determine crucial histone modifications for Treg development and function and to study compounds able to revert or modify epigenetic patterns. 

Among histones modifications is acetylation, which occurs at lysine residues mainly on their amino-terminal tails. This posttranslational modification is dynamic and its overall effect on gene expression depends on the balance between histone acetyltransferases (HATs) and histone deacetylases (HDACs) [7, 8]. HDACs typically dampen histone-DNA and histone-non histone protein interactions [9, 10], but they also regulate the function of non-histone proteins [11].

Histone deacetylase inhibitors (HDACi) such as trichostatin A (TSA) are small molecule compounds capable of inhibiting class I, II, and IV HDAC families of enzymes [12]. Previous studies in mice have shown that TSA administration *in vivo* promotes the generation and function of Tregs [13], and has beneficial effects in cardiac allograft transplant, inflammatory bowel disease [13] and lupus models [14]. Unfortunately, none of these studies provided *in vitro* data regarding the effect of TSA on Treg generation from conventional CD4^+^CD25^−^ T cells and their suppressive function, making it difficult to understand whether these are direct or indirect effects. Our study provides evidence that TSA increases the generation of CD4^+^Foxp3^+^ Tregs *in vitro* and gains insight into the regulation of CD4^+^Foxp3^+^ Tregs by the deacetylase inhibitor TSA.

## 2. Materials and Methods

### 2.1. Animals

Six- to eight-week-old Foxp3-GFP mice were used for all experiments. All mice were maintained and manipulated according to institutional guidelines at the pathogen-free facility of Fundación Ciencia & Vida after approval by the Ethical Review Committee.

### 2.2. Antibodies

Anti-CD3*ε*, anti-CD16/32, anti-CD4-APCH7, anti-CD25-APC, anti-IL-17A-PE, anti-IFN*γ*-PECy7, and anti-CD39-PE, anti-GARP-PE, anti-CD73-Cy7 were purchased from eBioscience (CA, USA). Anti-CTLA-4-PE was purchased from BD PharMingen (NJ, USA). Anti-IL-10-PE, anti-LAG3-biotin, streptavidin-APC, anti-IL4, and anti-IFN*γ* were purchased from BioLegend (CA, USA). Anti-H3ac and anti-H3 were purchased from Abcam (MA, USA) and Rabbit IgG from Millipore (MA, USA).

### 2.3. Flow Cytometry and Cytokine Secretion Analysis

The expression of cell surface markers on T cells was determined by FACS analysis after surface staining with specific anti-mouse antibodies. To determine IL-10 secretion, Treg cells were stimulated for 4 h at 37°C with 50 ng/mL PMA and 1 ug/mL ionomycin (Sigma-Aldrich). Following the reactivation, the supernatants were recovered and immediately analyzed using a mouse BD cytometric assay array (CBA). All data were collected on a FACSCanto II (BD Biosciences) and analyzed with FACS Diva software (BD, New Jersey) or FlowJo software (TreeStar).

### 2.4. Purification of T Cells and *In Vitro* T Cell Differentiation

Splenic CD4^+^ T cells from Foxp3-GFP mice were enriched by MACS purification using the CD4 isolation kit II (Miltenyi Biotec) following the manufacturer's instructions. Naive CD4^+^CD25^−^ T cells were further purified by cell sorting using a FACS ARIA II (Becton Dickinson, NJ, USA). For T helper cell cultures, dendritic cells were enriched by MACS purification using the CD11c microbeads (Miltenyi Biotec). Naive CD4^+^ T cells were cocultured with dendritic cells at a 5 : 1 ratio, in the presence of 1 *μ*g/mL anti-CD3, 10 *μ*g/mL anti-IL-4, 10 *μ*g/mL anti-IFN-*γ* and the presence or absence of 10 nM TSA, under polarizing conditions towards iTregs (5 ng/mL TGF-*β*, 100 U/mL IL-2, and 10 nM retinoic acid) or Th17 cells (5 ng/mL TGF-*β*, 10 ng/mL IL-6, and 10 ng/mL IL-1*β*) for 4 days. Before harvesting for chromatin immunoprecipitation (ChIP), naive CD4^+^ and iTreg cells were purified by cell sorting after surface staining with anti-CD4 and anti-CD25. Cell sorting was gated on the CD4^+^CD25^−^Foxp3^−^ (naive) or CD4^+^Foxp3^+^ population (iTregs). For Th17 sorting, cells were stained with anti-CD4 and permeabilized for further anti-IL-17A staining. Sorting was gated on the CD4^+^IL17A^+^ cells.

### 2.5. Isolation of Splenic DCs

Spleen tissue was fragmented and digested for 45 min at 37°C in the presence of collagenase D (Roche, Germany) and 2 *μ*g/mL of DNAse I (Roche) in PBS plus 10% fetal bovine serum. Undigested fibrous material was removed by filtration through cell strainer. CD11c^+^ cells were obtained by positive selection using anti-CD11c microbeads (Miltenyi Biotec) according to the manufacturer's instructions.

### 2.6. Western Blot Analysis

Cells were washed twice with cold phosphate-buffered saline (PBS) and then lysed in RIPA buffer (50 mM Tris, 150 mM NaCl, 0.1% SDS, 0.5% Na deoxycholate, and 1% NP40) supplemented with a protease inhibitor cocktail. Protein concentration from lysates was determined by Bradford assay (Pierce, Rockford, IL, USA). Equal protein amount of cell lysates was loaded on the gels and subjected to SDS-PAGE. The separated proteins were transferred onto PVDF membranes and then analyzed by Western blotting using anti-H3 and anti-H3ac antibodies. Bands were quantified using Quantity One software (Bio-Rad).

### 2.7. ChIP and DNA Quantification

After cell sorting, cells were cross-linked with 1% formaldehyde for 10 min. The reaction was quenched with 0.125 M glycine for 5 min, and cells were washed twice with PBS, resuspended in cell lysis buffer (10 mM Tris-HCl pH 8.0, 10 mM NaCl, 0.2% NP-40, and a proteinase inhibitor cocktail) for 10 min, and then resuspended in nuclear lysis buffer (50 mM Tris-HCl pH 8.0, 10 mM EDTA, 1% SDS, and a proteinase inhibitor cocktail). Lysate was sonicated with a Branson Sonifier 250, diluted twice in sonication buffer (50 mM HEPES pH 7.9, 140 mM NaCl, 1 mM EDTA, 1% Triton X-100, 0.1% deoxycholic acid, 0.1% SDS, and a proteinase inhibitor cocktail), and precleared with rabbit IgG and protein A agarose (Millipore). 3 ug of the precleared samples was taken as input control and for ChIP reactions, which were performed overnight at 4°C with 1 ug of anti-H3ac or 1 ug of rabbit IgG. Immunocomplexes were isolated with protein A agarose and washed twice with the sonication buffer, once with wash buffer (500 mM LiCl, 100 mM Tris HCl pH 8.0, 1% NP-40, and 0.1% deoxycholic acid), and once with TE (50 mM Tris-HCl pH 8.0 and 2 mM EDTA). Samples were then eluted with 100 uL of the immunoprecipitation elution buffer (50 mM NaHCO_3_ and 1% SDS), and finally the cross-link was reversed by incubating the samples at 65°C in 200 mM NaCl. Samples were treated with 1 mg/mL proteinase K and the DNA isolated by DNA Clean and Concentrator kit (Zymo Research). For quantification of the immunoprecipitated DNA, KAPA SYBR FAST (KAPA Biosystems) was used according to the manufacturer's instructions. Immunoprecipitated DNA was quantified by creating a line of best fit from a standard curve using serial dilutions of genomic template DNA to allow normalization of primer sets. Primers for the *foxp3* promoter were as follows: forward, 5′ CCTTGGCAACATGATGGTGGTGAT 3′; reverse, 5′ AAGAAGGGATCAGAAGCCTGCCAT 3′.

### 2.8. Analysis of Treg Cell Function

For Treg suppression assays, carboxyfluorescein-succinimidyl-ester- (CFSE-, Invitrogen) labeled effector T cells (CD4^+^CD25^−^) were stimulated with 1 mg/mL of antibody against CD3*ε* (eBioscience) in the presence of CD11c^+^ dendritic cells at a ratio of 1 : 2 (T effector: dendritic cells) and at a ratio of 1 : 0.5 of T effector: purified CD4^+^Foxp3^+^ Treg cells generated with or without TSA. To rule out that TSA acted upon dendritic cells rather than on the T lymphocytes, suppression assays were performed after activating the T cells in the presence of plate-bound anti-CD3 (0.5 *μ*g/mL and 5 *μ*g/mL) and soluble anti-CD28 (1 *μ*g/mL) and in the absence of dendritic cells. After 72 hours, suppression of proliferation was determined by flow cytometry analysis of CFSE dilution using a FACS Canto II cytometer (BD), and data were analyzed using FACS Diva software (BD).

## 3. Results

### 3.1. The *foxp3* Promoter Is Hyperacetylated on iTreg Cells

**Figure 1:**
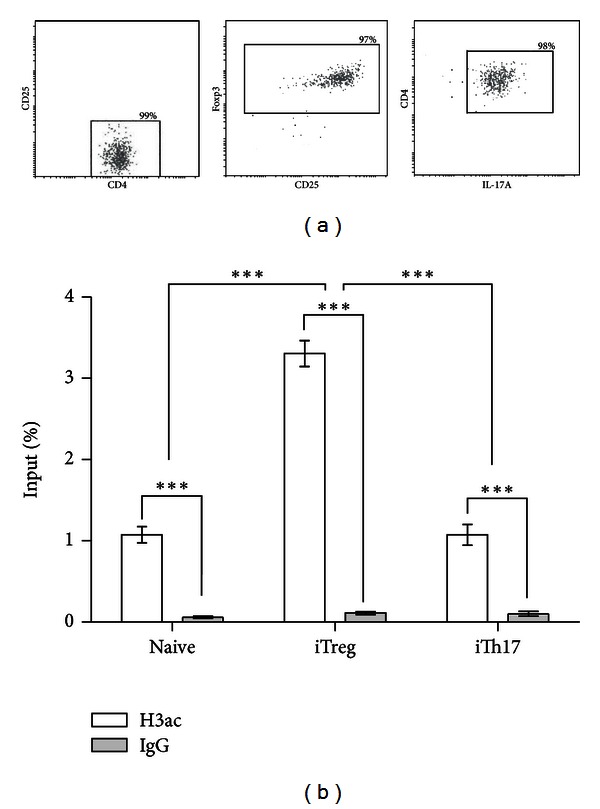
The *foxp3* promoter is hyperacetylated on iTreg cells. (a) Flow cytometry of the sorted naive cells and *in vitro* differentiated Treg and Th17 cells. Numbers indicate the percentage of naive (CD4^+^CD25^−^), Treg (CD4^+^Foxp3^+^), and Th17 (CD4^+^IL-17^+^) positive cells. (b) chromatin immunoprecipitation assays on *foxp3* promoter. ChIP assays were performed on sorted splenic naive CD4^+^ T cells and *in vitro* induced Tregs and Th17 cells. DNA fragments bound to acetylated histones were immunoprecipitated using antibodies directed against acetylated histone H3 (H3ac) or a rabbit isotype-matched immunoglobulin G (IgG), as control. Precipitated DNA was quantified by real-time PCR with primers specific for the *foxp3* gene promoter, and the PCR products were set in relation to input DNA. Standard deviation was obtained from three (naive and iTregs) and two (iTh17) independent experiments.

We first sought to analyze the patterns of histone H3 acetylation on differentiated Treg cells. For this purpose, we cultured naive (CD4^+^CD25^−^Foxp3^−^) T cells with spleen-derived CD11c^+^ DC plus *α*-CD3 mAb under Treg polarizing conditions for 4 days and then cells were sorted to purify the resulting Treg population (CD4^+^CD25^+^Foxp3^+^) (Figure 1(a)). We then analyzed by ChIP assay the H3ac mark on the histone H3 of the *foxp3* promoter on both, the sorted naive CD4^+^ T cells and the *in vitro* iTreg (Figure 1(b)). The results show that when compared to the naive CD4^+^ T cells, histone H3 is hyperacetylated at the *foxp3* promoter in iTreg cells. To demonstrate that the acetylation of the *foxp3* promoter was related to the differentiation of naive CD4^+^ T lymphocytes to Treg cells, we also compared the level of H3ac of the *foxp3* promoter on *in vitro* differentiated naive CD4^+^ T cells to Th17 cells (Figure 1(a)). As expected, the acetylation levels of the *foxp3* promoter did not change on differentiated Th17 cells. Thus, we conclude that the *foxp3* promoter becomes hyperacetylated on histone H3 upon differentiation of naive CD4^+^ T cells to Treg cells.

### 3.2. TSA Increases the Generation of Treg *In Vitro*

**Figure 2:**
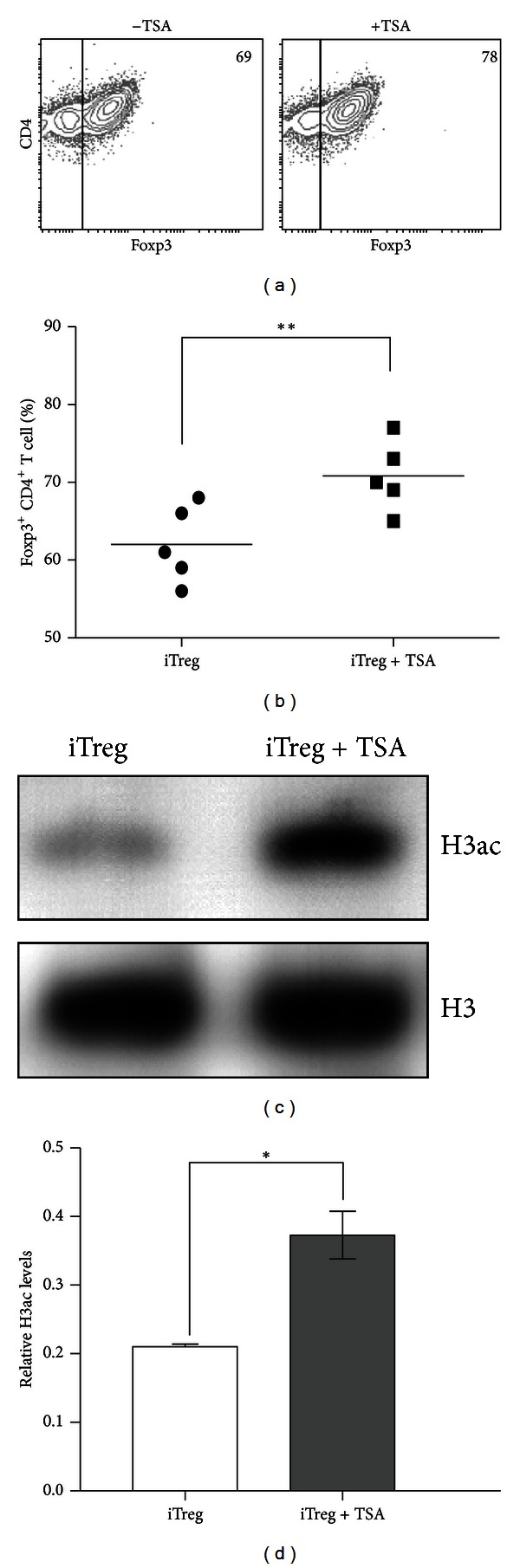
TSA increases the generation of Tregs from naive CD4^+^ T cells. (a) Flow cytometry analysis of CD4^+^Foxp3^+^ cells generated in the absence (left) or presence (right) of 10 nM TSA for 4 days. Numbers indicate the percentage of CD4^+^Foxp3^+^ double positive cells. (b) Scatter plot depicts percentage of CD4^+^Foxp3^+^ double positive cells from five independent experiments in the presence and absence of TSA. **P < 0.008 by student's *t*-tests. (c) Western blot analyses of total protein extracts derived from Tregs differentiated in the absence or presence of 10 nM TSA, as indicated. (d) Quantitation of the acetylation levels relative to the histone H3 with the Quantity One software. Standard deviation was obtained from three independent experiments. *P < 0.05 by two-way ANOVA.

Since the acetylation patterns of histone H3 changes during Treg differentiation, we next questioned whether by affecting the acetylation levels of histone H3, Treg differentiation could be pharmacologically modulated. For this purpose, we cultured naive (CD4^+^CD25^−^Foxp3^−^) T cells with spleen-derived CD11c^+^ dendritic cells plus *α*-CD3 mAb under iTreg polarizing conditions in the presence or absence of the histone deacetylase inhibitor TSA. After 4 days, Foxp3-GFP expression was assessed by FACS, as an indicator of Treg differentiation.  Figure 2(a) shows that 69% of the population expressed Foxp3, whereas the percentage increased to 78% when naive T cells were differentiated in the presence of TSA. Statistical analyses on 6 independent experiments indicated that the TSA treatment increased the differentiation of Tregs by 14.8% compared to untreated cultures (Figure 2(b)). To confirm that the mechanism involved in the effect of TSA on Treg differentiation implied changes on the acetylation levels of histone H3, we isolated total protein extracts from iTreg and analyzed the acetylation levels of histone H3. Western blot analyses showed that TSA treatment increased the global levels of acetylated histone H3 on Tregs by nearly two fold compared to untreated cultures (Figures 2(c) and 2(d)). Taken together, these results suggest that TSA stimulates the differentiation of Tregs while at the same time increasing the levels of histone H3 acetylation.

### 3.3. TSA Upregulates Treg Suppressive Capacity

**Figure 3:**
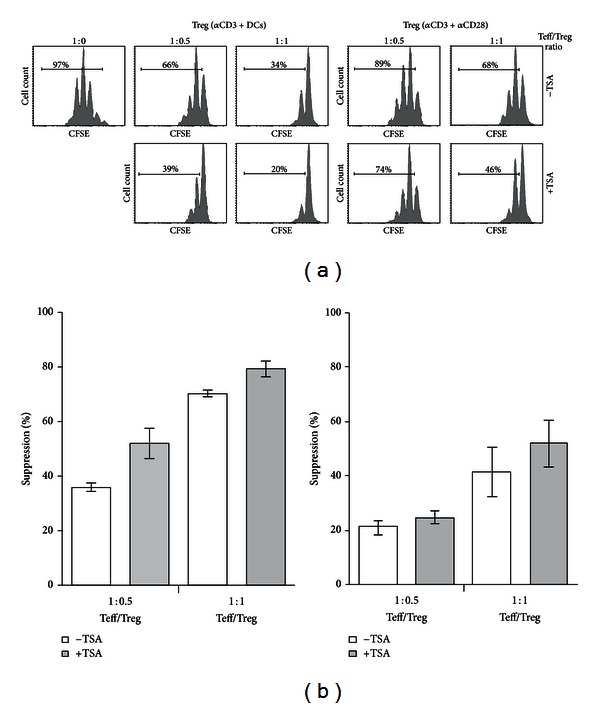
TSA Treg presents higher suppressive capacity. (a) Treg suppression assays of Tregs generated in the presence (left) or absence (right) of dendritic cells, and with (+TSA) or without TSA (−TSA). Tregs from each group were added to CFSE-labeled naive CD4^+^ T cells cocultivated with dendritic cells plus anti-CD3 antibody. The percentage of proliferating T cells is shown in each plot. Data are representative of three independent experiments. (b) Quantitation of the percentage of suppression of Tregs generated with or without TSA in the presence of anti-CD3 and dendritic cells (left) or plate-bound anti-CD3 and soluble anti-CD28 (right).

Next we tested the activity of the Tregs generated in the presence of TSA. For this, CD4^+^Foxp3^+^ Treg cells were generated as indicated above and after 4 days of culture the resultant Foxp3^+^CD4^+^ population was cocultured with CFSE-labeled naive T cells in the presence of CD11c^+^ DCs and anti-CD3, in a conventional T cell suppression assay.  Figure 3 shows that at a ratio of 1 effector to 0.5 Treg, the proliferation of the naive T lymphocytes was clearly suppressed by the Treg. Interestingly, Treg generated in the presence of TSA showed a higher suppressive activity compared to those generated in the absence of the drug. Therefore, these results show that TSA increased the ability of Tregs to suppress the *in vitro* proliferation of CFSE-labeled naive CD4^+^ T cells. To determine if the effect of TSA on the activity of Tregs was due to a direct effect of the drug on Treg cells, Tregs were generated in the absence of dendritic cells by activating naive T cells with plate-bound anti-CD3 and soluble anti-CD28. The results show that TSA increases the suppressive activity of Tregs even when generated in the absence of dendritic cells (Figures 3(a) and 3(b)). However, Tregs generated in the presence of dendritic cells show higher suppressive activity than those generated using activating antibodies. Moreover, the number of Tregs generated in the presence of dendritic cells needed to achieve 50% suppression is half of those generated in the presence of activating antibodies (ratio 1 : 0.5 in the presence of dendritic cells versus 1 : 1 in the presence of activating antibodies).

### 3.4. Tregs Generated under TSA Treatment Differentially Express Treg Markers Involved in Their Suppressive Functions

**Figure 4:**
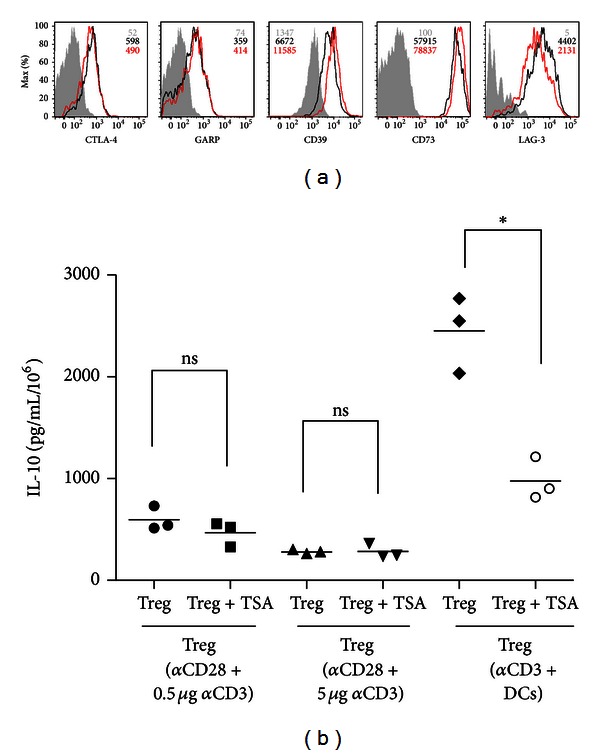
Phenotypical characterization of Tregs generated under TSA treatment. (a) Representative histograms of CTLA-4, GARP, CD39, CD73, and LAG-3 expression on Tregs generated in the absence (black) or presence (red) of TSA. Controls are filled histograms. Average MFI ± SEM was derived from three independent experiments. (b) IL-10 secretion was determined using the CBA method. Each point represents an individual mouse.

Besides Foxp3 and IL-10 secretion, several additional markers such as CTLA-4 (cytotoxic T-lymphocyte associated molecule-4), GITR (glucocorticoid-induced TNF receptor), and LAG-3 are also expressed on regulatory T cells; however the functional significance of these molecules remains to be defined. Also the ectonucleotidases CD39 and CD73 are expressed in Treg and convert ATP into immunosuppressive adenosine. As TSA increased the differentiation and suppressive functions of Treg cells, we decided to analyze whether TSA was capable of inducing changes in the expression of these additional Treg markers. For this purpose, we generated Tregs in the presence or absence of TSA for 4 days as described, and we then stained the cells with specific antibodies.  Figure 4 shows that although no significant changes could be detected in the expression of CTLA-4 or GARP, the mean fluorescence of TSA-treated Treg for the ectonucleotidases CD39 and CD73 showed significant increase of 1.7- and 1.4-fold, respectively. We also observed a modest (0.5-fold) decrease in the mean fluorescence of LAG-3. These results indicate that TSA may affect the immune suppressive activity of Treg by specifically increasing the protein levels of the ectonucleotidases CD39 and CD73, the expression of which is driven by Foxp3, the Treg-specific transcription factor. This result may be a clue to the mode of action of TSA on Treg activity. When analyzing IL-10 secretion, we found no differences in Tregs generated in the presence or absence of TSA, when these Tregs were generated in the absence of dendritic cells. However, we found that TSA produced higher IL-10 secretion (2.5 fold) when Tregs were produced in the presence of DCs, probably due to an effect of TSA on DCs rather than on the Tregs.

## 4. Discussion

It is well known that histone posttranslational modifications play an important role in the regulation of gene expression and that pharmacological control of histone-modifying enzymes could be potentially used to manipulate the differentiation of specific cellular lineages. On the other hand, defects in the development or function of regulatory T cells contribute significantly to the pathogenesis of many inflammatory and autoimmune diseases. Thus, there are important attempts to develop new strategies for generating Foxp3^+^ regulatory T cells as a valuable therapy for these diseases. HDAC inhibitors have previously shown to induce cell-cycle arrest and apoptosis in cancer and CD4^+^ T cells [15, 16] and to have anti-inflammatory effects on human monocytes [17]. Specifically, the histone deacetylase inhibitor TSA has been shown to modulate inflammatory and immune responses by boosting thymic production of naturally occurring Treg cells and also to increase the suppressive function of Treg *in vivo* [13].

In this study, we analyzed the effect of TSA on the differentiation of naive CD4^+^ T cells towards a regulatory phenotype and performed a functional and phenotypic characterization of TSA-differentiated Tregs. We observed that TSA stimulates the differentiation of naive T cells towards a Treg phenotype and that this stimulation correlated with hyperacetylation of the global histone H3. This suggests that TSA may act through the hyperacetylation of the histone H3 in the *foxp3* promoter. Interestingly, it has been reported that the Foxp3 protein becomes acetylated as well, preventing its proteasomal degradation [18]. Thus, the increased CD4^+^Foxp3^+^ T cell population produced by TSA could possibly be the result of combined pathways: by the upregulation of the *foxp3* gene expression mediated by the hyperacetylation of the histone H3 on the *foxp3* promoter and, on the other hand, by increasing the Foxp3 protein's half-life. The stimulation of Treg differentiation that we observed by TSA could explain, at least in part, the anti-inflammatory effect of TSA. This could be due to an increase in peripheral Treg cells generation, as it has been suggested by Tao et al. [13]. 

Since our differentiation experiments were carried out in the presence of CD11c^+^ dendritic cells we wanted to rule out that TSA affected the DC and thus indirectly Treg activity. Our results show that Tregs generated in the absence of DC but in the presence of activating antibodies also show an increased suppressive activity, indicating that TSA acts on naive T cells. However the results also suggest that dendritic cells may become more tolerogenic in the presence of TSA, since the number of Tregs generated in the presence of TSA and dendritic cells needed to achieve 50% suppression is half of those generated in the presence of activating antibodies (ratio 1 : 0.5 in the presence of dendritic cells versus 1 : 1 in the presence of activating antibodies). Consistently, it has been reported that TSA can change dendritic cells into a tolerogenic phenotype *in vitro* [19], so the evidence points to the fact that both Tregs and dendritic cells are targets for TSA.

Importantly, we also demonstrated that TSA increases Treg functions. Tregs exert their functions in several ways. The widely recognized mechanisms of suppression include the secretion of suppressive soluble factors, cell contact-mediated suppression, and competition for growth factors. The phenotypic characterization of Tregs generated in the presence or absence of TSA gave us some lights on the mechanisms by which TSA could be affecting Treg suppressive functions. We found that TSA increased the expression of CD39 and CD73 ectonucleotidases in Tregs. These ectoenzymes generate pericellular adenosine from extracellular nucleotides that suppress effector T cell functions [20]. The correlation between the TSA effect and the level of these ectoenzymes could explain the higher suppressive activity observed in Treg cells generated under the TSA treatment. In contrast, TSA decreased the expression of LAG-3, a molecule involved in Treg cell suppression of dendritic cell function [21], suggesting that the expression of CD39 and CD73 might compensate this reduction. On the other hand, no differences in the expression of GARP or CTLA-4 were observed between both types of Treg cells. Although we found that TSA could increase the production of IL-10, these effects could only be seen in the condition where Tregs were generated in the presence of dendritic cells, so it could be due to an effect on them. Further work needs to be performed to better understand the Treg cells suppressor mechanisms being affected by TSA. A better understanding of the effect of TSA on Treg cells should provide important tools to therapeutically enhance Treg production and suppressive function. In conclusion, our results showed that the outcome of pharmacological modulation of histone-modifying enzymes on the differentiation of Tregs might be exploited as potential valuable therapy for autoimmune diseases.
